# Optimisation of the Conversion and Extraction of Arctigenin From *Fructus arctii* Into Arctiin Using Fungi

**DOI:** 10.3389/fmicb.2021.663116

**Published:** 2021-05-31

**Authors:** Zheng Lu, Bin He, Jie Chen, Li-Jun Wu, Xia-Bing Chen, Sheng-Qiang Ye, Wen-Hai Yang, Zhi-Yong Shao, Er-Guang Jin, Si-Jiu Wang, Hong-Bo Zhou, Ji-Yue Cao

**Affiliations:** ^1^College of Veterinary Medicine, Huazhong Agricultural University, Wuhan, China; ^2^Institute of Animal Husbandry and Veterinary, Wuhan Academy of Agricultural Sciences, Wuhan, China; ^3^College of Veterinary Medicine, Northwest Agriculture & Forestry University, Yangling, China

**Keywords:** *Fructus arctii*, arctiin, arctigenin, microbial fermentation, preparation process

## Abstract

*Fructus arctii* is commonly used in Chinese medicine, and arctiin and arctigenin are its main active ingredients. Arctiin has low bioavailability in the human body and needs to be converted into arctigenin by intestinal microbes before it can be absorbed into the blood. Arctigenin has antiviral, anti-inflammatory, and anti-tumour effects and its development has important value. In this study, we used external microbial fermentation with *Aspergillus awamori* and *Trichoderma reesei* to process and convert arctiin from *F. arctii* powder into arctigenin, hence increasing its bioavailability. We developed a fermentation process by optimising the carbon and nitrogen source/ratio, fermentation time, pH, liquid volume, inoculation volume, and substrate solid-liquid ratio. This allowed for an arctiin conversion rate of 99.84%, and the dissolution rate of the final product was 95.74%, with a loss rate as low as 4.26%. After the fermentation of *F. arctii* powder, the average yield of arctigenin is 19.51 mg/g. Crude fermented *F. arctii* extract was purified by silica gel column chromatography, and we observed an arctigenin purity of 99.33%. Our technique effectively converts arctiin and extracts arctigenin from *F. arctii* and provides a solid basis for further development and industrialisation.

## Introduction

*Fructus arctii*, also known as great power seed, was originally described in “The Miscellaneous Records of Famous Physicians.” It is derived from the dry and ripe fruit of *Arctium lappa* L., a biennial herb of the Compositae family, and is commonly used in Chinese medicine, which is cold, pungent, and with bitter taste. It has cooling properties on the lung and stomach meridians. It disperses wind-heat, detoxifies rashes, and diminishes swelling and promotes healing lung rashes. *F. arctii* is mainly used to treat sore throat, fever, cough, and rashes and was even used to treat mumps, rubella, carbuncle boils, syphilis, and scurvy with similar uses in veterinary medicine in the past (Chen, 2015; Gao et al., 2015). *F. arctii* is mainly used to treat sore throat, fever, cough, and rashes and, in the past, was even used to treat mumps, rubella, carbuncle boils, syphilis, and scurvy (Chen, 2015) with similar uses in veterinary medicine (Gao et al., 2015). The main active components are lignans, including arctiin and arctigenin. Arctiin has low bioavailability but can be converted into arctigenin by gastrointestinal microorganisms (Hu et al., 2004; Yu, 2007; He et al., 2019). Modern science has demonstrated that arctigenin is beneficial for the treatment of viruses (Swarup et al., 2008; Hayashi et al., 2010), tumours (Matsumoto et al., 2006; Kim et al., 2010), inflammation (Zhao et al., 2009), allergy (Lee and Kim, 2010), shock (Ishihara et al., 2006), bacteria (Liu, 2008), and parasites (Wang et al., 2009). Furthermore, it has immunomodulatory (Cho et al., 1999), neuroprotective (Jang et al., 2002), hepatoprotective (Kim et al., 2003), and anti-diabetic effects (Lu, 2007). Therefore, it is a traditional Chinese herb that warrants development.

The natural content of arctigenin in *F. arctii* is only about 0.19%, while the content of arctiin can reach 3.5% (Ming et al., 2004). Arctiin has low bioavailability in humans and needs to be converted into arctigenin by intestinal microbial enzymolysis before it can be absorbed into the blood (Hu et al., 2004; Yu, 2007). Hu et al. (2004) demonstrated that a snail enzyme could be used for the enzymatic digestion of arctiin into arctigenin. The use of β-glucosidase yields high conversion rates of arctiin into arctigenin (Zhang et al., 2012).

In this study, we optimised the process of enzymatic hydrolysis of arctiin *in vitro* using fungal strains known for their high expression of β-glucosidase. Six fungi and different culture conditions were used to ferment *F. arctii* powder to break the glycosidic bond of arctiin and convert it into arctigenin. We then used a new method to separate and purify arctiin from the fermented material by silica gel column chromatography. Thus, we established a simple and efficient method that could eventually be upscaled for the industrialisation of arctigenin conversion into arctiin. This method was patented in China (#CN103233056A).

## Materials and Methods

### Study Design

In this study, three parameters were used to determine the performance of the fermentation conditions: the conversion rate of arctiin, sample dissolution rate, and sample loss rate. First, six fungal strains were tested alone or in combination to determine the best yield of arctiin. Then, the best fungus or combination was tested in different conditions. Each condition (carbon source, nitrogen source, culture volume, culture time, and inoculation volume) was first tested alone. Then, based on the outcomes, an orthogonal design was used to determine the best combination of different conditions.

### Preparation of the Fungal Strains

The ingredients listed in Supplementary Table 1 were added to form 1,000 mL of Mandel nutrient solution, and the pH was adjusted to 5.0–6.0 with sulphuric acid. The solution was sterilised at 115°C for 20 min and stored for later use.

We sterilised 8.0 g *F. arctii* powder (200 mesh), 5.0 g of bran, 5.0 g of cornflour, and 0.6 g of peptone at 121°C for 15 min. Then, 20 mL of sterile Mandel nutrient solution was added, and the volume of the solution was adjusted to 130 mL with sterile water and sterilised at 121°C for 15 min.

*Aspergillus phoenicis*, *Aspergillus awamori*, *Aspergillus oryzae*, *Aspergillus niger*, *Trichoderma viride*, and *Trichoderma reesei* lyophilised fungal powder were purchased from the China Industrial Microbial Culture Collection Management Center. The fungi were evenly spread on a potato dextrose agar (PDA) plate and cultured at 28–30°C for 5–6 days until mature spores formed. Then, we added sterile water to dilute the spore suspension to a concentration of 10^6^–10^7^ units/mL. We inoculated 2.0 mL of spore suspension in 50 mL of Mandel nutrient solution and cultured the fungi at 175 rpm and 28–30°C for 1–2 days to obtain the seed solutions (one for each fungus).

### Fermentation of *Fructus arctii* Powder

The six seed solutions were inoculated in triplicate in the fermentation medium, according to Supplementary Table 2, at 30°C and 150 rpm for 168 h. The samples were dried at 100°C, and the dry matter was pulverised and diluted in 50 mL of methanol. After sonification for 20 min and overnight rest, the solution was filtered and evaporated to obtain the crude fermented *F. arctii* extract.

### Determination of Arctiin and Arctigenin

The crude fermented *F. arctii* extract and the arctiin and arctigenin standard solutions were analysed by thin-layer chromatography (TLC). The developing agent was a mix of chloroform, methanol, and glacial acetic acid in a 95:5:0.15 ratio. The strain with the most darkened arctigenin spots was used as the primary fermentation strain for the subsequent experiments.

We used an Agilentl1200 high-performance liquid chromatograph (HPLC) with a ZORBAX Extend-C18 column (4.6 mm × 250 mm, 5 μm). The mobile phase of the HPLC was methanol and water in a volume ratio of 67:33 with a flow rate of 1.0 mL/min. The injection volume was 10 μL. The column temperature was maintained at 30°C, and the detection wavelength was 280 nm (Ju, 2008; He et al., 2019).

We dissolved 9.18 mg of arctiin standard (purity ≥ 99.8%) and 23.36 mg of arctigenin standard (purity ≥ 99.8%) in 50 mL of methanol to prepare a mixed standard solution. We made serial dilutions of the standard solution in methanol and used HPLC to analyse the solutions. The arctiin standard (A_1_) and arctigenin standard (A_2_) peak areas were used to calculate arctiin (C_1_) and arctigenin (C_2_) concentration in the *F. arctii* powder and crude fermented *F. arctii* extract.

To determine the linear regression between arctiin and arctigenin, 2, 4, 6, 8, and 10 mL of the mixed standard solution of arctiin and arctigenin was accurately measured in a 10-mL volumetric flask and diluted with methanol. Arctiin and arctigenin were determined as above. Linear regression was performed with the measured arctiin peak area (A_1_) and the arctiin concentration (C_1_) to calculate the regression equation. The same was performed for the arctigenin peak area (A_2_) versus the arctigenin concentration (C_2_).

The conversion rate of arctiin, sample dissolution rate, and sample loss rate were calculated by the following formulas: T = (C_01_-C_e__1_)/C_01_ × 100; S = (C_e__1_ + C_e__2_)/(C_01_ + C_02_) × 100; L = 100 – S; where T = conversion rate, S = dissolution rate, L = loss rate, C_01_ = arctiin concentration in unfermented powder, C_e__1_ = arctiin concentration in fermented powder, C_02_ = arctigenin concentration in unfermented powder, and C_e__2_ = arctigenin concentration in fermented powder. The selection criteria for the final fermentation strain were a conversion rate > 99%, dissolution rate > 95%, and sample loss rate < 5%.

### Fermentation Optimisation

Based on the arctiin conversion rate, sample dissolution rate, and loss rate, we examined the fermentation of the strains using different carbon or nitrogen sources, liquid loading, pH, and culture time to optimise the fermentation conditions.

Carbon sources: corn flour (C_1_), bran (C_2_), sucrose (C_3_), bran and corn flour (C_4_), bran and sucrose (C_5_), sucrose and corn flour (C_6_), and bran and sucrose and corn flour (C_7_).

Nitrogen sources: peptone (N_1_), soybean meal (N_2_), urea (N_3_), ammonium sulfate (N_4_), ammonium nitrate (N_5_), and ammonium chloride (N_6_).

Culture volumes: 30, 50, 70, 90, 110, 130, and 150 mL.

pH: 4, 5, 6, 7, 8, 9, and 10.

Culture times: 36, 48, 72, 96, 120, 144, and 168 h.

Inoculation volume: 1.0, 2.0, 3.0, 4.0, and 5.0 mL.

Each condition (carbon source, nitrogen source, culture volume, culture time, and inoculation volume) was first tested alone. Then, based on the outcomes, we designed an orthogonal experiment with five following factors at four different levels: *F. arctii* powder quantity, seed inoculation ratio, carbon to nitrogen ratio, Mandel nutrient solution volume, and solid–liquid ratio (Supplementary Table 3). The solid–liquid ratio was the ratio of the amount of solid matter (e.g., *F. arctii* powder, bran, and peptone) to the amount of liquid (e.g., Mandel nutrient solution and water). For each condition used in the orthogonal design, the top conditions in terms of conversion, dissolution, and sample loss rates were used. All combinations of the four conditions were tested, yielding 16 different groups of combinations. The experiments were performed in triplicate, and the arctigenin output rate was measured and averaged.

### Determination of Enzymatic Activity

The β-glucosidase activity was measured in all conditions (groups 1–22, Supplementary Table 2) at 36 and 168 h using the β-Glucosidase Activity Assay Kit from Sigma-Aldrich (St Louis, MO, United States), according to the manufacturer’s instructions. The β-glucosidase activity is expressed as U/mL, and 1 U is defined as the production of 1 μmol of glucose per mL of enzyme solution per minute. pNPGase, cellulase, and xylanase activities were measured using commercial kits (Sigma-Aldrich, St Louis, MO, United States), according to the manufacturer’s instructions.

### Purification of the Fermented Extract and Arctigenin Purity Determination

We wet-packed a 30 cm × 2 cm column with 40 g of silica gel (200–300 mesh) using chloroform. The crude fermented *F. arctii* extract was dissolved until saturation in 2.0 mL chloroform and injected in the column for purification. The eluent was chloroform and ethyl acetate in a 10:2 volume ratio. We used a rotary evaporator to collect the eluent under reduced pressure and dried the product, which was further purified (as described above) a second time. The eluent was collected and dried, and crystallised arctigenin was obtained using methanol. HPLC (as described above) was used to determine the amount of arctigenin in the sample.

### Statistical Analysis

The results are presented as means ± standard deviations and were analysed using ANOVA and Tukey’s *post hoc* test. All analyses were performed using SPSS 16.0 (IBM, Armonk, NY, United States). Two-sided *P*-values <0.05 were considered statistically significant.

## Results

### Strain Screening

**FIGURE 1:**
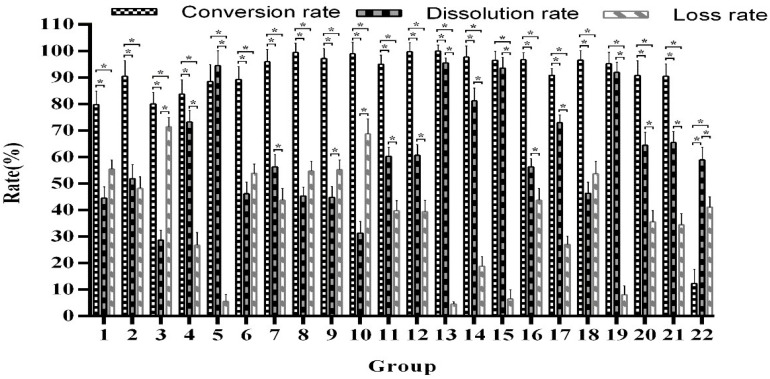
Comparison of the single and mixed strain fermentation capabilities. *Indicates significance at the 0.01 level.

Thin-layer chromatography showed that the selected strains and combinations of strains all could convert arctiin. Subsequently, all sample extracts were quantitatively tested by HPLC to determine the best fermentation strain. We observed that many combinations have strong fermentation ability, with arctiin conversion rates of more than 95.05%. After a comprehensive analysis, we found that the best conversion rate is obtained from *T. reesei* combined with *A. awamori* (Figure 1, Group 13), which reached 98.92–99.95%, with a dissolution rate of 94.63–96.45%. The comparison of the fermentation of different strains and their combinations is shown in Figure 1. The β-glucosidase activity is shown in Supplementary Table 4.

### Optimization of the Fermentation Conditions

**FIGURE 2:**
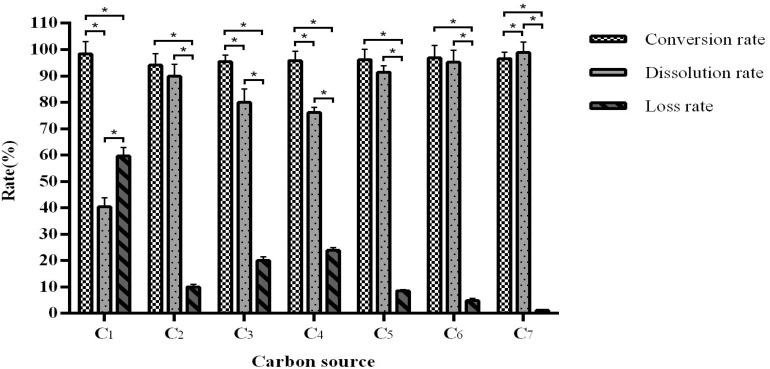
The influence of the carbon source on fermentation efficiency. *Indicates significance at the 0.01 level.

Different carbon sources do not affect the conversion rate, which was maintained between 94.09 and 98.45%, but it does have a significant effect on the dissolution and loss rates (Figure 2). We observed that mixing equal amounts of bran, sucrose, and cornflour as a carbon source results in the most optimal fermentation efficiency with a conversion rate of 94.31–98.45% and dissolution rate of 95–99.61%.

**FIGURE 3:**
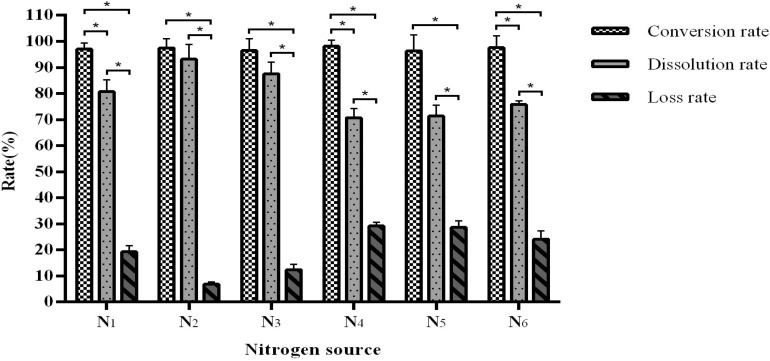
The influence of the nitrogen source on fermentation efficiency. *Indicates significance at the 0.01 level.

Similar to the carbon source data, different nitrogen sources have no significant effect on the conversion rate (96.43–98.08%) but affected the dissolution and loss rates (Figure 3). We observed that when using urea as a nitrogen source, the fermentation conversion rate reaches an optimal 96.58–98.08% and with a dissolution rate of 92.91–96.53%.

**FIGURE 4:**
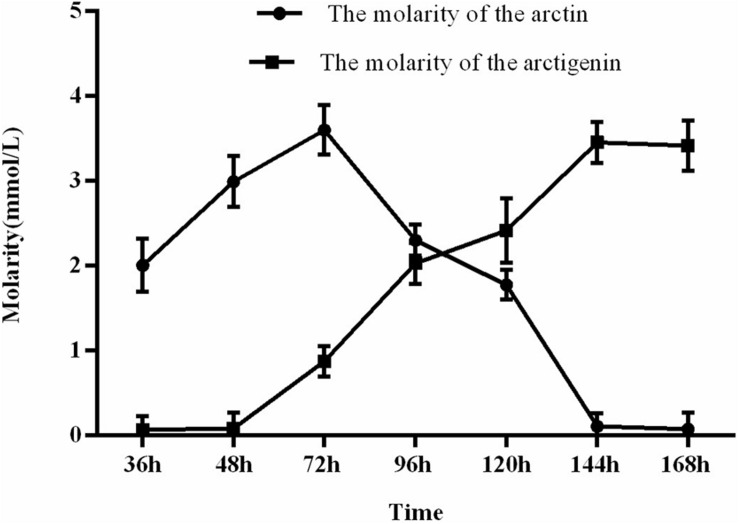
Concentration of arctiin and arctigenin over time during the fermentation process.

As expected, the fermentation time affected the concentrations of arctiin and arctigenin as the fungi converted the substrates (Figure 4). After 144 h of fermentation, there is no significant change in the concentration of arctiin and arctigenin, suggesting an ideal fermentation time.

**FIGURE 5:**
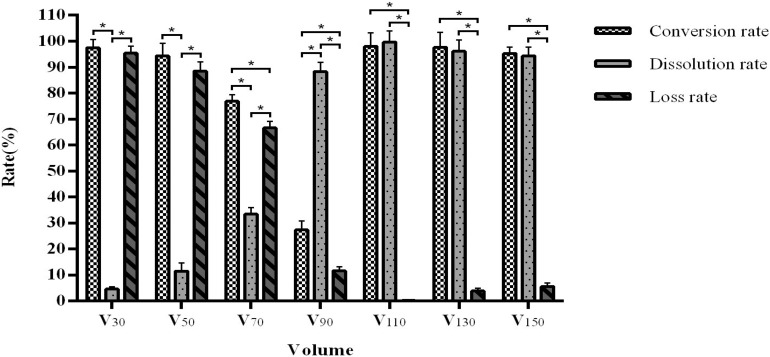
The influence of medium volume on fermentation efficiency. *Indicates significance at the 0.01 level.

Interestingly, we observed that when the volume is lowered, the arctigenin yield is extremely low due to a high loss rate. An increase of volume is associated with an increased dissolution rate with an optimal rate above 90 mL, although the conversion rate shows a downward trend. When the volume reaches 110 mL, further increases do not affect the product conversion rate (96.93–98.65%) and dissolution rate (98.65–99.78%) (Figure 5), suggesting an ideal fill volume of 110 mL.

**FIGURE 6:**
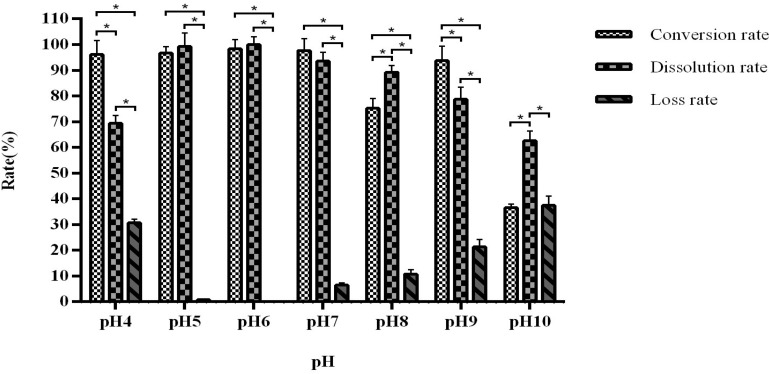
The influence of pH on fermentation efficiency. *Indicates significance at the 0.01 level.

When the pH is maintained between 5 and 7, the fermentation conversion rate is between 96.68 and 98.34% (Figure 6). The optimal is pH 6, with a dissolution rate of 98.57–99.89%.

**FIGURE 7:**
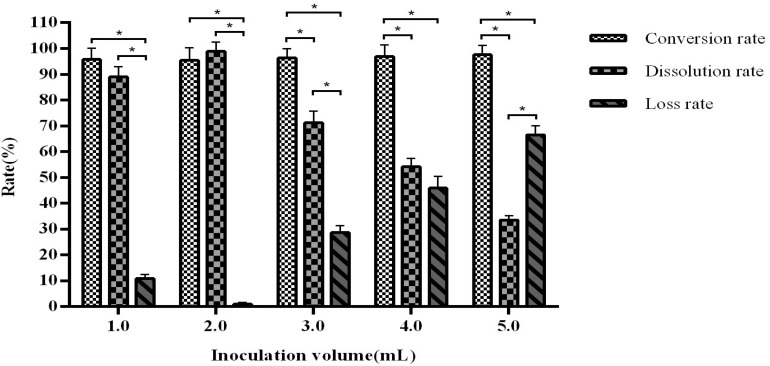
The influence of inoculation volume on fermentation efficiency. *Indicates significance at the 0.01 level.

When the inoculation volume is greater than 3 mL, the product dissolution rate decreases with correlating increases in the loss rate. An inoculation volume of 2 mL in the previously established fermentation volume of 110 mL was found to be optimal, with a conversion rate of 94.54–96.76% and dissolution rate of 97.65–99.61% (Figure 7).

**FIGURE 8:**
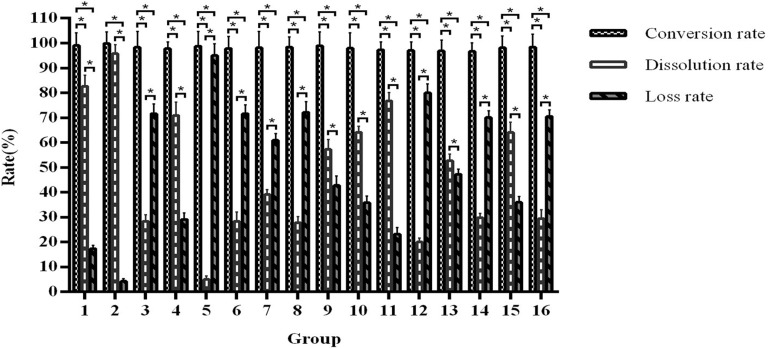
Orthogonal optimisation of fermentation conditions. *Indicates significance at the 0.01 level.

After these single factor optimisation tests, we further optimised the fermentation conditions by orthogonal testing. As shown in Figure 8, the product conversion rate of all test groups is 96.63–99.84%. Different *F. arctii* powder quantity, seed inoculation ratio, carbon to nitrogen ratio, Mandel nutrient solution volume, and solid–liquid ratio have a great influence on the dissolution rate. According to the results, the fermentation is the most effective in the conditions of group 2, which shows a conversion rate of 97.56–99.84% and a dissolution rate of 94.56–96.78%.

In addition, the maximum arctigenin output in group 2 is 19.51 ± 0.98 mg/g of *F. arctii* powder (Supplementary Table 5). The factors that influence this yield are *F. arctii* powder quantity, solid–liquid ratio, Mandel nutrient solution volume, seed inoculation ratio, and carbon to nitrogen ratio, ranked from high to low. The optimal fermentation conditions are 52% *F. arctii* powder in the fermentation substrate, 1:2 seed inoculation ratio of *A. awamori* and *T. reesei*, 100:6 carbon to nitrogen ratio, 10 mL of Mandel nutrient solution in 110 mL total volume, and 1:2 solid-liquid ratio. Supplementary Table 6 presents the pNPGase, cellulase, and xylanase activities of the optimal condition.

### Purification of Arctigenin and Purity Determination

After purification, we used HPLC as described above to determine the amount of arctigenin in the sample. We determined that the purity of arctigenin is between 99.14 and 99.52% (Table 1). A linear regression analysis was performed on the arctiin concentration (C_1_) and peak area (A_1_). The regression equation was A_1_ = 2.3 × 10^5^ × C_1_ + 1588.4 with r = 0.9997. Arctiin concentration has a good linear relationship with the peak area within the concentration range of 0.068–0.340 mmol/L. Similarly, arctigenin concentration (C_2_) shows a good linear relationship with the peak area (A_2_) in the concentration range of 0.286–1.430 mmol/L with the regression equation A_2_ = 5.3 × 10^6^ × C_2_ + 1.4 × 10^6^ and r = 0.9992.

**TABLE 1:** Arctigenin purity after purification.

Sample	Sample concentration (mg/mL)	Arctigenin concentration (mg/mL)	Arctigenin purity (%)
1	1.051	1.042 ± 0.004	99.14
2	1.053	1.046 ± 0.006	99.34
4	1.049	1.044 ± 0.001	99.52

## Discussion

The natural content of arctigenin in *F. arctii* is only about 0.19%, while the content of arctiin can reach 3.5% (Ming et al., 2004). In this study, we screened different fermentation conditions *in vitro* that simulate the intestinal microbial environment using the joint fermentation of different fungal strains with high production of β-glucosidase to directly prepare arctigenin from *F. arctii*. The method is simple, practical, and inexpensive, and is suitable for industrial upscaling.

Hu et al. (2004) demonstrated that the enzymatic digestion of arctiin with a snail enzyme yielded a conversion rate to arctigenin of 72%. The use of β-glucosidase boosts the conversion rates to up to 90.94% (Zhang et al., 2012). There are many sources of β-glucosidase, such as in animals, fruits, and microorganisms (Yang et al., 2013; Mallek-Fakhfakh and Belghith, 2016). Plant-derived β-glucosidase activity is lower than those from microorganisms such as bacteria, filamentous fungi, actinomycetes, and yeast; in particular, *Archaea*, *Aspergillus*, *Penicillium*, *Trichoderma*, and *Arthrobacter* can produce a large amount of β-glucosidase with high activity (Uchiyama et al., 2015; Albaser et al., 2016). Arctiin is normally transformed into arctigenin in the digestive tract by the β-glucosidase from enterobacteria, but the use of enterobacteria for the production of a compound for medicinal use might pose some problem regarding the presence of lipopolysaccharides. In addition, fungi produce higher amounts of β-glucosidase than enterobacteria (Uchiyama et al., 2015; Albaser et al., 2016). Screening for high-yield and highly active glucosidase is crucial for achieving an efficient system for the biotransformation of arctiin (Yang et al., 2015; Chang et al., 2018). Therefore, in this study, six strains of fungi with high-yield β-glucosidase were screened. The results showed that the combination of *A. awamori* and *T. reesei* yielded the highest efficiency for transforming arctiin into arctigenin. No previous studies are available for comparison, but the combination of those two fungi has been shown to be effective in various industrial processes that require the digestion of cellulose and β-glucosidase activity (Poutanen et al., 1986; Friedrich et al., 1987; Makhatov et al., 2019).

The optimisation of the fermentation conditions is critical to the production process. Factors such as fermentation substrate composition and time have a significant impact on efficiency and may even lead to changes in the product itself. The optimal conditions are (1) equal amounts of bran, sucrose, and cornflour as the carbon source, (2) urea as the nitrogen source, (3) fermentation time of 144 h, (4) fermentation volume of 110 mL, (5) fermentation pH of 6, (6) inoculation volume of 2 mL per 110 mL fermentation liquid, (7) fermentation substrate consisting of 52% *F. arctii* powder, (8) 1:2 inoculation ratio of *A. awamori* and *T. reesei*, (9) 100:6 carbon-nitrogen ratio, (10) 10 mL Mandel nutrient solution per 110 mL fermentation liquid, and (11) 1:2 solid–liquid ratio.

Some carbon and nitrogen sources stimulate the production of other enzymes to assist in decomposing and dissolving arctiin and arctigenin. Inversely, other sources may counteract the production of cellulase and hinder the decomposition of the cell wall, leading to insoluble arctiin and a decline in fermentation yield. The concentration of arctiin increases in the medium from 0 to 72 h, and then decreases. Arctiin can be bound to the cell wall of *F. arctii* cells (Gao et al., 2018). Since *A. awamori* and *T. reesei* can produce cellulase (Friedrich et al., 1987), the bound arctiin is released with the degradation of the cell wall, leading to an increase in arctiin concentration over the first 72 h of culture. After 72 h, the cell walls are digested and the β-glucosidase activity predominates, leading to the decrease in arctiin concentrations until its near-complete digestion into arctigenin. Therefore, 144 h is considered as the optimal fermentation duration.

The fermentation volume mainly affects the growth space of the fungal strain and the dissolved oxygen content in the liquid. We observed that when the volume is in the range of 30–90 mL, the dissolution rate increases with increasing volume, but the conversion rate shows a downward trend. This may be due to an unbalanced output of various enzymes from the fermentation strains, which are unable to meet the conversion requirements. Interestingly, our data demonstrated that an inoculation volume of greater than 3 mL reduces the dissolution rate and increases the loss rate. This may be explained by the limited amount of nutrients in the fermentation system. When the inoculation volume is too large, it is likely that the nutritional supply will be insufficient, resulting in abnormal growth and enzyme production of the fungi, which affects the yield. We further optimised our process through orthogonal testing and achieved an arctiin conversion rate of more than 99%.

We used a microbial co-fermentation method to directly prepare arctigenin from *F. arctii*. Previously reported conversion rates using *A. niger* was 92.3–94.7% (Ou et al., 2011, 2019). Other studies using high-yield enzyme strains achieved conversion rates of 90–96% (Bai, 2006; Xu, 2007; Huang, 2014). A common theme between these studies is the use of a single fermenting strain. In comparison, our joint fermentation using two strains is more conducive to the transformation of arctiin, with significantly higher conversion rates. This indicates that the production of β-glucosidase may be higher, and the activity may be stronger during co-fermentation. It is possible that the two selected strains of fungi have a mutually beneficial symbiotic relationship that promotes growth and increases enzyme production during the fermentation process.

Studies have investigated organic methods to extract arctiin from *F. arctii* and have shown extraction rates of up to 94.98% (Wang et al., 2003). Furthermore, alcohol extraction and silica gel column chromatography to separate and purify arctiin and arctigenin allow for 95.5 and 94% purity, respectively (Lu, 2011). A mixture of degreased fruit powder of *F. arctii* and 50% alcohol solution containing 5% hydrochloric acid was refluxed for 5 h. and then *F. arctii* was extracted with chloroform twice. The crude extract was purified by silica gel column chromatography resulting in about 99.3% arctigenin, and the approximately 1.1% total yield (Ye et al., 2011). Arctigenin was extracted from fruits of *F. arctii* powder by supercritical carbon dioxide, The percent content of arctigenin was about 99.66%, and the total yield was approximately 0.45% (Yang et al., 2007). In the optimal extraction process herbs meal were taken, plus 1.5 times the amount of water soaking herbs for 8 h, and then 10 times amount of water extracting three times with 1 h each time, The transfer rate of arctiin was 67% (Gong and Gao, 2010). We applied microbial fermentation and silica gel column chromatography to convert, separate, and purify arctigenin from *F. arctii* powder without additional separation and extraction enzymes. Using our methods, the conversion rate of arctiin to arctigenin is as high as 99.84%, and the yield of arctigenin from 1 g of *F. arctii* powder is 19.51 mg under the optimal conditions. Silica gel column chromatography was used to purify arctigenin, and our methods improve the purity of arctigenin to 99.33%,with the total yield approximately 1.95%. According to the current market sales of *F. arctii* 3 US dollars/kg, and Arctigenin Standard,3 US dollars/mg, the cost of arctigenin obtained by this method is relatively high, very low. Thus, we have established a simple and efficient method for preparing arctigenin from *F. arctii*, which can be applied in the industrial development of *F. arctii* and related products and promote modernisation of traditional Chinese medicine in China. At the same time, as *F. arctii* and its main active ingredient arctigenin have good anti-virus, anti-inflammatory and many other pharmacological effects, it is believed that in the near future, arctigenin and its processing products will make a positive contribution to people’s life and health in the world.

## Conclusion

A combination of *A. awamori* and *T. reesei* is the best fungal combination to convert arctiin in *F. arctii* powder to arctigenin. The optimal fermentation conditions were also determined, achieving arctiin conversion rates of 99.84% with dissolution rates of 95.73% and loss rates of 4.26%, yielding 19.51 mg arctigenin per 1 g *F. arctii* powder. This study demonstrates an effective method to extract arctigenin from *F. arctii* that can be upscaled for the industrialisation of arctigenin as a potential new drug with antiviral, anti-tumour, and anti-inflammatory benefits.

## Data Availability Statement

The original contributions presented in the study are included in the article/Supplementary Material, further inquiries can be directed to the corresponding author.

## Author Contributions

ZL and BH: experimental study and manuscript writing. JC, L-JW, X-BC, S-QY, Z-YS, and W-HY: experimental study. S-JW, H-BZ, and E-GJ: data analysis. J-YC: experimental design and revising the manuscript. All authors contributed to the article and approved the submitted version.

## Conflict of Interest

The authors declare that the research was conducted in the absence of any commercial or financial relationships that could be construed as a potential conflict of interest.

## References

[B1] Albaser A. Kazana E. Bennett M. (2016). Discovery of a bacterial glycoside hydrolase family 3 (GH3)β-glucosidase with myrosinase activity from a citrobacter strain isolated from soil. *J. Agricul. Food Chem.* 64 1520–1527. 10.1021/acs.jafc.5b05381 26820976

[B2] Bai J. F. (2006). *Studies on Microbial Transformation of Fructus arctii.* Chengdu: Sichuan University.

[B3] Chang Z. S. Lan H. Bao Y. L. Liu Z. Y. (2018). Progress of β-glucosidase from microorganisms. *Adv. Microbiol.* 7 79–86. 10.12677/amb.2018.72010

[B4] Chen D. (2015). *Pharmacopoeia Commission of the People’s Republic of China.* Beijing: China medical science and technology press.

[B5] Cho J. Y. Kim A. R. Yoo E. S. Baik K. U. Park M. H. (1999). Immunomodulatory effect of arctigenin, a lignan compound, on tumour necrosis factor-alpha and nitric oxide production, and lymphocyte proliferation. *J. Pharm. Pharmacol.* 51 1267–1273. 10.1211/0022357991777001 10632084

[B6] Friedrich J. Cimerman A. Perdih A. (1987). Mixed culture of *Aspergillus awamori* and *Trichoderma reesei* for bioconversion of apple distillery waste. *Environ. Microbiol.* 26 299–303. 10.1007/bf00286328

[B7] Gao H. B. Yu K. Z. Zhang Z. Q. Fong Z. W. Xu X. J. Xia X. Z. (2015). *People’s Republic of China Veterinary Pharmacopoeia.* Beijing: China Agriculture Press.

[B8] Gao Q. Yang M. Zuo Z. (2018). Overview of the anti-inflammatory effects, pharmacokinetic properties and clinical efficacies of arctigenin and arctiin from *Arctium lappa* L. *Acta Pharmacol. Sin.* 39 787–801. 10.1038/aps.2018.32 29698388 PMC5943914

[B9] Gong Y. M. Gao N. (2010). Reseach on extration methods of arctinin. *Clin. Med. Eng.* 17 63–64.

[B10] Hayashi K. Narutaki K. Nagaoka Y. Hayashi T. Uesato S. (2010). Therapeutic effect of arctiin and arctigenin in immunocompetent and immunocompromised mice infected with influenza A virus. *Biol. Pharm. Bull.* 33 1199–1205. 10.1248/bpb.33.1199 20606313

[B11] He B. Zhang H. J. Yang W. H. (2019). Pharmacokinetics of arctigenin and *Fructus arctii* powder in piglets. *Front. Vet. Sci.* 6:235. 10.3389/fvets.2019.00235 31403047 PMC6669357

[B12] Hu Y. J. Fan Y. H. Xiao M. X. Zhou J. Lu Y. Y. Yang Z. F. (2004). Study on the preparation of aglycone from arctiin hydrolyzed by snail enzyme. *J. Guangzhou Univ. Trad. Chin. Med.* 21 473–475.

[B13] Huang G. (2014). *Study on Biotransformation and Characterization of Glycosides Hydrolase from Arctium.* Wyhan: Huazhong University of Science and Technology.

[B14] Ishihara K. Yamagishi N. Saito Y. Takasaki M. Konoshima T. Hatayama T. (2006). Arctigenin from *Fructus arctii* is a novel suppressor of heat shock response in mammalian cells. *Cell Stress Chaperones* 11 154–161. 10.1379/csc-148r.1 16817321 PMC1484516

[B15] Jang Y. P. Kim S. R. Choi Y. H. Kim J. Kim S. G. Markelonis G. J. (2002). Arctigenin protects cultured cortical neurons from glutamate-induced neurodegeneration by binding to kainate receptor. *J. Neurosci. Res.* 68 233–240. 10.1002/jnr.10204 11948668

[B16] Ju M. J. (2008). *Study on Extraction Technology of Lignans from Arctium lappa L.* Shunyang: Liaoning University of Traditional Chinese Medicine.

[B17] Kim J. Y. Hwang J. H. Cha M. R. Yoon M. Y. Son E. S. Tomida A. (2010). Arctigenin blocks the unfolded protein response and shows therapeutic antitumor activity. *J. Cell. Physiol.* 224 33–40. 10.1002/jcp.22085 20232300

[B18] Kim S. H. Jang Y. P. Sung S. H. Kim C. J. Kim J. W. Kim Y. C. (2003). Hepatoprotective dibenzylbutyrolactone lignans of *Torreya nucifera* against CCl4-induced toxicity in primary cultured rat hepatocytes. *Biol. Pharm. Bull.* 26 1202–1205. 10.1248/bpb.26.1202 12913279

[B19] Lee J. Y. Kim C. J. (2010). Arctigenin, a phenylpropanoid dibenzylbutyrolactone lignan, inhibits type I-IV allergic inflammation and pro-inflammatory enzymes. *Arch. Pharm. Res.* 33 947–957. 10.1007/s12272-010-0619-1 20607501

[B20] Liu K. (2008). In vitro antibacterial experiment of Fructus Arctium lappa decoction, *Arctium aglycone* and *Arctium aglycone*. *Tianjin Pharm.* 20 10–11.

[B21] Lu C. L. (2007). *Study on the Seperation and purification way of arctiin and Study on the protective Mechanism Vascular Endothelial Cell of Experimental Diabetic Rats with Arctiin.* Chongqing: Army Medical University.

[B22] Lu K. X. (2011). Extraction and purification of arctiin and arctigenin. *Zhejiang Chem. Indus.* 42 6–8.

[B23] Makhatov Z. Kedelbayev B. Lieberzeit P. Dzhakashyeva M. Elemanova Z. Abildayeva R. (2019). Biosynthesis of cellulase with *Trichoderma viride* and *Aspergillus awamori* micromycetes in co-cultivation. *Eur. J. Biosci.* 13 1521–1526.

[B24] Mallek-Fakhfakh H. Belghith H. (2016). Physicochemical properties of thermotolerant extracellular beta-glucosidase from *Talaromyces thermophilus* and enzymatic synthesis of cello-oligosaccharides. *Carbohydr. Res.* 419 41–50. 10.1016/j.carres.2015.10.014 26649918

[B25] Matsumoto T. Hosono-Nishiyama K. Yamada H. (2006). Antiproliferative and apoptotic effects of butyrolactone lignans from *Arctium lappa* on leukemic cells. *Planta Med.* 72 276–278. 10.1055/s-2005-916174 16534737

[B26] Ming J. Y. Wang Z. C. Song C. Q. (2004). Quantitative analysis of arctiin and arctigenin of *Arctium lappa* L. from different areas by HPLC *Lishizhen*. *Med. Mater. Med. Res.* 15 737–739.

[B27] Ou Z. M. Duo Z. H. Shi H. B. (2011). Study on the transformation of arctiin from water extract of arctiin from aspergillus Niger. *Chin. Trad. Herbal Drugs* 4 698–700.

[B28] Ou Z. M. Yan Q. Y. Yang G. S. (2019). Preparation of arctigenin by hydrolysis of arctii by biotransformation method. *J. Zhejiang Univ. Technol.* 37 629–633.

[B29] Poutanen K. Puis J. Linko M. (1986). The hydrolysis of steamed birchwood hemicellulose by enzymes produced by *Trichoderma reesei* and *Aspergillus awamori*. *Appl. Microbiol. Biotech.* 23 487–490. 10.1007/bf02346065

[B30] Swarup V. Ghosh J. Mishra M. K. Basu A. (2008). Novel strategy for treatment of Japanese encephalitis using arctigenin, a plant lignan. *J. Antimicrob. Chemother.* 61 679–688. 10.1093/jac/dkm503 18230688

[B31] Uchiyama T. Yaoi K. Miyazaki K. (2015). Glucose-tolerant beta-glucosidase retrieved from a Kusaya gravy metagenome. *Front. Microbiol.* 6:548. 10.3389/fmicb.2015.00548 26136726 PMC4468940

[B32] Wang G. X. Han J. Feng T. T. Li F. Y. Zhu B. (2009). Bioassay-guided isolation and identification of active compounds from *Fructus arctii* against *Dactylogyrus intermedius* (Monogenea) in goldfish (*Carassius auratus*). *Parasitol. Res.* 106 247–255. 10.1007/s00436-009-1659-7 19859737

[B33] Wang X. L. Zhang Y. J. Shi R. B. (2003). The extraction technology of arctium lappa L. was optimized by orthogonal experiment. *J. Beijing Univ. Trad. Chin. Med.* 26 64–65.

[B34] Xu F. Y. (2007). *Theoretical and Applied Studies on Microbial Transformation of Fructus arctii.* Chengdu: Sichuan University.

[B35] Yang F. Yang X. Li Z. Du C. Wang J. Li S. (2015). Overexpression and characterization of a glucose-tolerant beta-glucosidase from T. aotearoense with high specific activity for cellobiose. *Appl. Microbiol. Biotechnol.* 99 8903–8915. 10.1007/s00253-015-6619-9 25957152

[B36] Yang H. Y. Cai S. Z. Zheng Y. M. (2007). Preparation of arctigenin from *Fructus Arctii* by two-step process. *Fine Chem.* 2 885–889.

[B37] Yang S. Hua C. Yan Q. Li Y. Jiang Z. (2013). Biochemical properties of a novel glycoside hydrolase family 1 beta-glucosidase (PtBglu1) from *Paecilomyces thermophila* expressed in *Pichia pastoris*. *Carbohydr. Polym.* 92 784–791. 10.1016/j.carbpol.2012.09.086 23218368

[B38] Ye S. Q. Chen J. Jin E. G. (2011). The extraction and purification of arctigenin. *Chin. J. Vet. Drug* 45 32–34.

[B39] Yu H. (2007). The chemical composition and biological activity of burdock. *W Phytomed.* 22 246–247.

[B40] Zhang L. Y. Yang Y. S. Zhang T. (2012). Research on the preparative method of arctigenin. *J. Chin. Med. Mat.* 35 467–470. 22876688

[B41] Zhao F. Wang L. Liu K. (2009). In vitro anti-inflammatory effects of arctigenin, a lignan from *Arctium lappa* L. through inhibition on iNOS pathway. *J. Ethnopharmacol.* 122 457–462. 10.1016/j.jep.2009.01.038 19429312

