# Supplementary material for: Optimisation of the Conversion and Extraction of Arctigenin From Fructus arctii Into Arctiin Using Fungi
Source: Front Microbiol. 2021 May 31;12:663116. doi: 10.3389/fmicb.2021.663116 (PMC8200475; doi:10.3389/fmicb.2021.663116)
Supplement: Supplementary file 1 [file Data_Sheet_1.docx]

# The ANOVA and Tukey test analysis result

# Table 1. P values for comparison between groups (Figure 1)

|  | ANOVA test | Tukey test | | |
| --- | --- | --- | --- | --- |
|  |  | Conversion rate vs. Dissolution rate | Conversion rate vs. Loss rate | Dissolution rate vs. Loss rate |
| Group |  |  |  |  |
| 1 | <0.001 | <0.001 | 0.001 | 0.053 |
| 2 | <0.001 | 0.001 | <0.001 | 0.759 |
| 3 | <0.001 | <0.001 | 0.016 | <0.001 |
| 4 | <0.001 | 0.024 | <0.001 | <0.001 |
| 5 | <0.001 | 0.280 | <0.001 | <0.001 |
| 6 | <0.001 | <0.001 | <0.001 | 0.258 |
| 7 | <0.001 | <0.001 | <0.001 | 0.032 |
| 8 | <0.001 | <0.001 | <0.001 | 0.053 |
| 9 | <0.001 | <0.001 | <0.001 | 0.036 |
| 10 | <0.001 | <0.001 | <0.001 | <0.001 |
| 11 | <0.001 | <0.001 | <0.001 | 0.002 |
| 12 | <0.001 | <0.001 | <0.001 | <0.001 |
| 13 | <0.001 | <0.001 | <0.001 | <0.001 |
| 14 | <0.001 | 0.003 | <0.001 | <0.001 |
| 15 | <0.001 | 0.425 | <0.001 | <0.001 |
| 16 | <0.001 | <0.001 | <0.001 | 0.003 |
| 17 | <0.001 | 0.001 | <0.001 | <0.001 |
| 18 | <0.001 | <0.001 | <0.001 | 0.231 |
| 19 | <0.001 | 0.446 | <0.001 | <0.001 |
| 20 | <0.001 | 0.002 | <0.001 | 0.001 |
| 21 | <0.001 | 0.002 | <0.001 | 0.001 |
| 22 | <0.001 | <0.001 | 0.005 | 0.042 |

# Table 2. P values for comparison between groups (Figure 2)

|  | ANOVA test | Tukey test | | |
| --- | --- | --- | --- | --- |
|  |  | Conversion rate vs. Dissolution rate | Conversion rate vs. Loss rate | Dissolution rate vs. Loss rate |
| Carbon source |  |  |  |  |
| C1 | <0.001 | <0.001 | <0.001 | <0.001 |
| C2 | <0.001 | 0.300 | <0.001 | <0.001 |
| C3 | <0.001 | 0.002 | <0.001 | <0.001 |
| C4 | <0.001 | <0.001 | <0.001 | <0.001 |
| C5 | <0.001 | 0.078 | <0.001 | <0.001 |
| C6 | <0.001 | 0.700 | <0.001 | <0.001 |
| C7 | <0.001 | 0.001 | <0.001 | <0.001 |

# Table 3. P values for comparison between groups (Figure 3)

|  | ANOVA test | Tukey test | | |
| --- | --- | --- | --- | --- |
|  |  | Conversion rate vs. Dissolution rate | Conversion rate vs. Loss rate | Dissolution rate vs. Loss rate |
| Nitrogen source |  |  |  |  |
| N1 | <0.001 | 0.033 | <0.001 | <0.001 |
| N2 | <0.001 | 0.346 | <0.001 | <0.001 |
| N3 | <0.001 | 0.189 | <0.001 | <0.001 |
| N4 | <0.001 | 0.001 | <0.001 | <0.001 |
| N5 | <0.001 | 0.003 | <0.001 | <0.001 |
| N6 | <0.001 | 0.001 | <0.001 | <0.001 |

# Table 4. P values for comparison between groups (Figure 5)

|  | ANOVA test | Tukey test | | |
| --- | --- | --- | --- | --- |
|  |  | Conversion rate vs. Dissolution rate | Conversion rate vs. Loss rate | Dissolution rate vs. Loss rate |
| Volume |  |  |  |  |
| V30 | <0.001 | <0.001 | 0.251 | <0.001 |
| V50 | <0.001 | <0.001 | 0.186 | <0.001 |
| V70 | <0.001 | <0.001 | 0.023 | <0.001 |
| V90 | <0.001 | <0.001 | 0.006 | <0.001 |
| V110 | <0.001 | 0.141 | <0.001 | <0.001 |
| V130 | <0.001 | 0.871 | <0.001 | <0.001 |
| V150 | <0.001 | 0.955 | <0.001 | <0.001 |

# Table 5. P values for comparison between groups (Figure 6)

|  | ANOVA test | Tukey test | | |
| --- | --- | --- | --- | --- |
|  |  | Conversion rate vs. Dissolution rate | Conversion rate vs. Loss rate | Dissolution rate vs. Loss rate |
| PH |  |  |  |  |
| pH1 | <0.001 | 0.001 | <0.001 | <0.001 |
| pH2 | <0.001 | 0.240 | <0.001 | <0.001 |
| pH3 | <0.001 | 0.103 | <0.001 | <0.001 |
| pH4 | <0.001 | 0.201 | <0.001 | <0.001 |
| pH5 | <0.001 | 0.016 | <0.001 | <0.001 |
| pH6 | <0.001 | 0.009 | <0.001 | <0.001 |
| pH7 | 0.001 | 0.001 | 0.969 | 0.001 |

# Table 6. P values for comparison between groups (Figure 7)

|  | ANOVA test | Tukey test | | |
| --- | --- | --- | --- | --- |
|  |  | Conversion rate vs. Dissolution rate | Conversion rate vs. Loss rate | Dissolution rate vs. Loss rate |
| Inoculation volume(mL) |  |  |  |  |
| 1 | <0.001 | 0.193 | <0.001 | <0.001 |
| 2 | <0.001 | 0.356 | <0.001 | <0.001 |
| 3 | <0.001 | 0.002 | <0.001 | <0.001 |
| 4 | <0.001 | <0.001 | <0.001 | 0.096 |
| 5 | <0.001 | <0.001 | <0.001 | <0.001 |

# Table 7. P values for comparison between groups (Figure 8)

|  | ANOVA test | Tukey test | | |
| --- | --- | --- | --- | --- |
|  |  | Conversion rate vs. Dissolution rate | Conversion rate vs. Loss rate | Dissolution rate vs. Loss rate |
| Group |  |  |  |  |
| 1 | <0.001 | <0.001 | <0.001 | <0.001 |
| 2 | <0.001 | 0.166 | <0.001 | <0.001 |
| 3 | <0.001 | <0.001 | <0.001 | <0.001 |
| 4 | <0.001 | <0.001 | <0.001 | <0.001 |
| 5 | <0.001 | <0.001 | 0.001 | <0.001 |
| 6 | <0.001 | <0.001 | <0.001 | <0.001 |
| 7 | <0.001 | <0.001 | <0.001 | <0.001 |
| 8 | <0.001 | <0.001 | <0.001 | <0.001 |
| 9 | <0.001 | <0.001 | <0.001 | <0.001 |
| 10 | <0.001 | <0.001 | <0.001 | <0.001 |
| 11 | <0.001 | <0.001 | <0.001 | <0.001 |
| 12 | <0.001 | <0.001 | <0.001 | <0.001 |
| 13 | <0.001 | <0.001 | <0.001 | 0.022 |
| 14 | <0.001 | <0.001 | <0.001 | <0.001 |
| 15 | <0.001 | <0.001 | <0.001 | <0.001 |
| 16 | <0.001 | <0.001 | <0.001 | <0.001 |

The concentration of Arctin and Arctigenin was determined by HPLC after fermentation result

Table 1. Concentrations of Arctin and Arctigenin after fermentation of single and mixed strains (Figure 1)

| Group | | Arctiin concentration(mg/mL) | | Arctigenin concentration(mg/mL) |
| --- | --- | --- | --- | --- |
| 1 | | 2.146 | 3.744 | |
| 2 | 5.889 | 6.272 | |  |
| 3 | | 2.112 | 1.668 | |
| 4 | | 1.721 | 7.971 | |
| 5 | | 6.470 | 1.184 | |
| 6 | | 4.016 | 5.694 | |
| 7 | | 4.315 | 7.015 | |
| 8 | | 6.728 | 5.930 | |
| 9 | | 4.278 | 1.258 | |
| 10 | | 1.201 | 4.003 | |
| 11 | | 5.296 | 7.409 | |
| 12 | | 4.144 | 7.979 | |
| 13 | | 4.278 | 1.258 | |
| 14 | | 1.049 | 1.072 | |
| 15 | | 6.364 | 1.231 | |
| 16 | | 1.426 | 6.027 | |
| 17 | | 9.884 | 8.648 | |
| 18 | | 5.912 | 6.071 | |
| 19 | | 1.036 | 1.205 | |
| 20 | | 1.192 | 7.318 | |
| 21 | | 1.019 | 7.643 | |
| 22 | | 1.517 | 6.267 | |

Table 2. Concentrations of Arctin and Arctigenin after fermentation with different carbon sources (Figure 2)

| Group | | Arctiin concentration(mg/mL) | Arctigenin concentration(mg/mL) | |
| --- | --- | --- | --- | --- |
| C1 | | 0.076 | | 1.451 |
| C2 | 0.290 | | 3.149 |  |
| C3 | | 0.222 | | 2.826 |
| C4 | | 0.205 | | 2.694 |
| C5 | | 0.188 | | 3.274 |
| C6 | | 0.157 | | 3.439 |
| C7 | | 0.166 | | 3.568 |

Table 3. Concentrations of Arctin and Arctigenin after fermentation with different nitrogen sources (Figure3)

| Group | | Arctiin concentration(mg/mL) | Arctigenin concentration(mg/mL) | |
| --- | --- | --- | --- | --- |
| N1 | | 0.145 | | 2.906 |
| N2 | 0.123 | | 3.387 |  |
| N3 | | 0.174 | | 3.143 |
| N4 | | 0.094 | | 2.571 |
| N5 | | 0.175 | | 2.537 |
| N6 | | 0.118 | | 2.745 |

Table 4. Concentrations of Arctin and Arctigenin after different fermentation time (Figure 4)

| Group | | Arctiin concentration(mg/mL) | Arctigenin concentration(mg/mL) | |
| --- | --- | --- | --- | --- |
| T36 | | 2.007 | | 0.067 |
| T48 | 2.992 | | 0.086 |  |
| T72 | | 3.604 | | 0.876 |
| T96 | | 2.302 | | 2.030 |
| T120 | | 1.779 | | 2.417 |
| T144 | | 0.109 | | 3.456 |
| T168 | | 0.076 | | 3.419 |

Table 5. Concentrations of Arctin and Arctigenin after fermentation with different liquid quantities (Figure 5)

| Group | | Arctiin concentration(mg/mL) | Arctigenin concentration(mg/mL) | |
| --- | --- | --- | --- | --- |
| V30 | | 0.124 | | 0.086 |
| V50 | 0.277 | | 0.234 |  |
| V70 | | 1.136 | | 0.453 |
| V90 | | 3.569 | | 0.805 |
| V110 | | 0.097 | | 3.647 |
| V130 | | 0.113 | | 3.502 |
| V150 | | 0.232 | | 3.353 |

Table 6. Concentrations of Arctin and Arctigenin after fermentation at different pH values (Figure 6)

| Group | | Arctiin concentration(mg/mL) | Arctigenin concentration(mg/mL) | |
| --- | --- | --- | --- | --- |
| PH4 | | 0.193 | | 2.451 |
| PH5 | 0.163 | | 3.582 |  |
| PH6 | | 0.082 | | 3.668 |
| PH7 | | 0.112 | | 3.406 |
| PH8 | | 1.218 | | 2.478 |
| PH9 | | 0.309 | | 2.717 |
| PH10 | | 3.117 | | 0.160 |

Table 7. Concentrations of Arctin and Arctigenin in fermentation after inoculation of different strains (Figure 7)

| Group | | Arctiin concentration(mg/mL) | Arctigenin concentration(mg/mL) | |
| --- | --- | --- | --- | --- |
| 1 | | 0.208 | | 3.175 |
| 2 | 0.226 | | 3.529 |  |
| 3 | | 0.179 | | 2.530 |
| 4 | | 0.150 | | 1.912 |
| 5 | | 0.120 | | 1.164 |

Table 8. The concentration of Arctin and Arctigenin after fermentation was optimized and combined (Figure 8)

| Group | | Combination | Arctiin concentration(mg/mL) | Arctigenin concentration(mg/mL) | |
| --- | --- | --- | --- | --- | --- |
| 1 | | 11111 | 0.047 | | 3.046 |
| 2 | 12222 | 0.106 | | 3.121 |  |
| 3 | | 13333 | 0.084 | | 0.997 |
| 4 | | 14444 | 0.110 | | 2.565 |
| 5 | | 21234 | 0.095 | | 0.233 |
| 6 | | 22143 | 0.166 | | 1.572 |
| 7 | | 23412 | 0.145 | | 2.225 |
| 8 | | 24321 | 0.126 | | 1.566 |
| 9 | | 31342 | 0.114 | | 4.629 |
| 10 | | 32431 | 0.227 | | 5.116 |
| 11 | | 33124 | 0.290 | | 6.110 |
| 12 | | 34213 | 0.320 | | 1.423 |
| 13 | | 41423 | 0.441 | | 5.228 |
| 14 | | 42314 | 0.465 | | 2.812 |
| 15 | | 43241 | 0.253 | | 6.542 |
| 16 | | 44132 | 0.218 | | 2.943 |
